# A Review on JC Virus Infection in Kidney Transplant Recipients

**DOI:** 10.1155/2013/926391

**Published:** 2013-01-29

**Authors:** Serena Delbue, Mariano Ferraresso, Luciana Ghio, Camilla Carloni, Silvia Carluccio, Mirco Belingheri, Alberto Edefonti, Pasquale Ferrante

**Affiliations:** ^1^Laboratory of Translational Research, Health Science Foundation “Ettore Sansavini”, Corso Garibaldi 11, 48022 Lugo, Italy; ^2^Department of General and Vascular Surgery, St. Joseph Hospital, Via San Vittore 12, 20122 Milan, Italy; ^3^Department of Clinical Sciences and Community Health, University of Milan, Via Francesco Sforza 35, 20122 Milan, Italy; ^4^Pediatric Nephrology and Dialysis Unit, IRCCS Ca' Granda Foundation “Ospedale Maggiore Policlinico”, Via Francesco Sforza 35, 20122 Milan, Italy; ^5^Laboratory of Translational Research, Department of Biomedical, Surgical and Dental Sciences, University of Milan, Via Pascal 36, 20133 Milan, Italy; ^6^Clinical Institute “Città Studi”, Via Ampere 47, 20133 Milan, Italy

## Abstract

The polyomavirus (PyV), JC virus (JCV), is a small nonenveloped DNA virus that asymptomatically infects about 80% of healthy adults and establishes latency in the kidney tissue. In case of immunodeficient hosts, JCV can lytically infect the oligodendrocytes, causing a fatal demyelinating disease, known as progressive multifocal leukoencephalopathy (PML). Although the reactivation of another human PyV, BK virus (BKV), is relatively common and its association with the polyomavirus associated nephropathy (PyVAN) following renal transplantation is proven, JCV replication and its impact on graft function and survival are less well studied. Here we describe the biology of JCV and its pathological features and we review the literature regarding the JCV infection analyzed in the setting of transplantations.

## 1. An Introduction to the Human Polyomavirus JC (JCV) 

JC virus (JCV) is a member of the Polyomaviridae family, including naked DNA viruses with icosahedral capsids and small, circular, and double-stranded DNA genomes. The natural hosts for polyomaviruses (PyVs) include humans, other primates, rodents, rabbits, and birds [1]. 

It was first isolated by Padgett et al. in 1971 from the brain of a patient with the initial JC, affected by Hodgkin's lymphoma who died of progressive multifocal leukoencephalopathy (PML), a demyelinating disease of the central nervous system (CNS) [2]. 

The 40–45 nm capsid is composed of three structural viral proteins, VP1, VP2, and VP3. The icosahedron consists of 72 pentamers, with no apparent hexamers, each composed by five VP1 molecules and one molecule of VP2 or VP3. The virion is formed of 88% protein and 12% DNA, represented by a single copy of supercoiled, circular, and double-stranded molecule of approximately 5.2 kb, associated with cellular histone proteins H2A, H2B, H3, and H4 and packaged into chromatin resembling cellular genomes (minichromosomes) [1, 3, 4].

The viral genome has a bipartite organization and contains two regions of about the same size, known as early and late transcription units, transcribed in opposite directions starting from a common hypervariable noncoding control region (NCCR), containing the origin of DNA replication (ori), the TATA box, cellular transcription factors binding sites, and bidirectional promoters and enhancers for the transcription of early and late genes. Starting from the NCCR, early transcription proceeds in a counterclockwise direction, while late transcription proceeds clockwise on the opposite strand of the DNA [5]. 

The early region spans 2.4 kb and encodes the alternatively spliced transforming proteins, large tumor antigen (T-Ag) and small tumor antigen (t-Ag), which are involved in the viral replication and in promoting transformation of cells in culture and oncogenesis in vivo [5]. 

**Figure 1:**
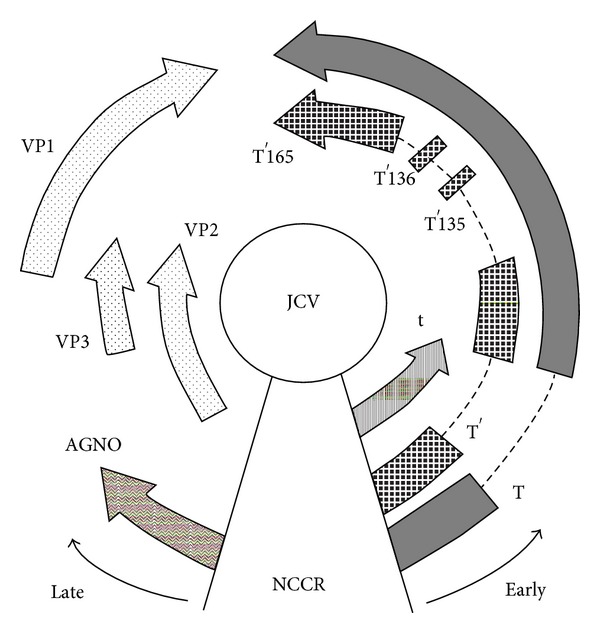
Schematic representation of the JCV genome organization. The circular, double-stranded DNA genome is ~5.2 kb in size and is divided into the early coding region and the late coding region, transcribed in opposite directions from a common noncoding control region (NCCR). Early genes include large T antigen (T-Ag), small t antigen (t-Ag), T′135, T′136, and T′165. Late genes include VP1, VP2, VP3, and agnoprotein.

The late coding region spans 2.3 kb and encodes the capsid proteins VP1, VP2, and VP3 by alternative splicing of a common mRNA derived from the same primary late transcript and a small regulatory protein, known as agnoprotein, whose function in the virus life cycle is not completely clear [5] (Figure 1).

JCV does not infect any species other than humans and its ability to infect human cells may be restricted at the level of viral early gene transcription and DNA replication, with the T-Ag interacting specifically with the human DNA polymerase [6].

JCV has a tropism for replication in human glial cells, kidney epithelial cells, and, with a less efficiency, in B lymphocytes, and the restricted CNS tropism is confirmed by both experimental animals and in vitro analysis [6, 7].

The transmission of JCV is not fully understood. JCV-specific antibodies are detected in approximately 70% of adults [8] and the primary infection occurs in early childhood, usually in an asymptomatic way and results in a primary viremia. Afterwards, the virus produces a persistent latent infection in the kidney and is shed into the urine. In the context of an immunosuppressive condition, such as AIDS and transplantation, JCV disseminates to the CNS and lytically infects oligodendrocytes, causing the demyelinating disease, known as PML [9–11].

## 2. Progressive Multifocal Leukoencephalopathy 

PML is a rare demyelinating disease characterized by the lytic infection of glial cells and is often fatal. The disease occurs almost exclusively in patients with severe immunodeficiency; consequently, the incidence of PML has increased dramatically, following the spread of HIV/AIDS. Nowadays, HIV infection is still the most frequent setting for PML, ~80% of the cases, followed by hematologic malignancies (~8%), solid cancers (~3%), organ transplantation, and autoimmune diseases treated with immunomodulators [12].

The classic form of PML is progressive and multifocal and involves the white matter. The main symptoms of the disease are motor deficits, altered consciousness, gait ataxia, and visual disturbances [13, 14]. Atypical presentations of PML are increasingly recognized and include cerebellar syndrome, reflecting productive infection of granule cell neurons [15], meningitis [16], meningoencephalitis [17, 18], progressive myoclonic ataxia [19] and muscle wasting associated to extrapyramidal signs [20]. The median survival of PML patients without HIV infection is of few months, while in HIV positive patients subjected to highly active antiretroviral therapy (HAART) is longer but characterized by severe impairment of CNS. Indeed, successful treatments for PML are not currently available. 

## 3. JCV and Transplantation: Epidemiology

Reports of JCV infection in renal transplant recipients have been published immediately after the first isolation of the virus [32, 33]. Since those times, subsequent works have investigated both the silent and the symptomatic infection and/or reactivation of JCV in the setting of kidney transplantation, finding contradictory results. In 1980, Hogan et al. reported active infection after transplantation with JCV in about half of the 61 patients investigated [33]. Gardner and colleagues performed a wide prospective, serological study for the evidence of JCV infection in forty-eight renal transplant recipients, finding that 54% of the patients were seropositive already before the operation and that in 23% of the seronegative patients JCV infection occurred within the first three months after transplantation [34]. Molecular analyses were also conducted, by means of specific hybridization in situ, PCR, and quantitative PCR assays by different international groups: JCV has been identified in kidney biopsy tissue and/or urine within a range of 3.4% and 46% of kidney transplanted patients (Table 1) [21–31, 35]. The most recent surveys, that had the possibility to measure the amount of replicating JCV in the clinical specimens, reported also a very wide range of viral loads, from 2.0 × 10^3^ copies/mL to 1 × 10^7^ copies/mL [21, 22, 25, 31, 35, 36]. The association between the JCV viruria and transplantation has not been proved yet. In fact, Yin and colleagues showed that JCV load was markedly increased in transplant patients compared to the healthy group, confirming the association between immune function and viral levels [25]. On the contrary, Husseiny and colleagues observed a very low level JCV shedding in the urine of both normal subjects (15%) and renal transplant recipients, with no differences among the viral loads [21]. Regarding JCV viremia in the renal transplant recipients, it seems to be very rare, transient, and low. In a cohort of 103 patients, it has been observed only in 14.2% of subjects with a mean viral load of 2 × 10^3^ copies/mL; in another study conducted on 20 patients, 25% showed JCV viremia from the lower limit of detection (of 25 copies/mL) up to 10^3^ copies/mL [21]. A long-term prospective follow-up study was conducted in France and JCV was detected in only 31 blood samples out of the 1487 collected [29].

Only few studies analyzed also the molecular features of the isolated virus, mainly observing that the JCV strains infecting the kidney transplantation recipients did not differ significantly from those infecting the immunocompetent subjects [36, 37]. On the contrary, Yin and colleagues observed a dramatic increase in the proportion of transplant patients carrying two or more genotype strains of JCV compared with control subjects assuming an association between uncommon JCV genotypes and immunosuppression [25].

Regarding the nonkidney solid organ transplants (SOT), the incidence and clinical manifestation of JCV infection have been even more poorly investigated. In 2005, two independent groups published very different results about JCV infection in liver transplant patients, reporting 1.7% and 22.7% of patients excreting the virus, respectively [35, 38]. More recently, Kusne and colleagues [39] examined the frequency of JCV urinary shedding in a longitudinal study on 41 kidney and 33 liver transplant recipients. The proportion of patients shedding JCV was found higher in the liver than in the kidney transplant group (71% versus 38%), with a viral load of 1.2 × 10^7^ copies/mL and 3.9 × 10^6^ copies/mL, respectively. In addition, none of the patients with JCV viruria had also JCV viremia.

A prospective prevalence study on 100 liver transplant children reported a JCV viruria in 19%, without clinical signs of viral infection. The viruria was not influenced by the extent of immunosuppressive therapy but it was found higher in pediatric liver transplant recipients [40] than reported in adult patients [41, 42].

Studies on the association between JCV infection and lung, pancreas and heart transplantations are very rare. Antonsson and colleagues [43] analyzed the seroprevalence and antibody stability of JCV in 441 organ transplant recipients, including 386 kidney, 9 heart, 1 kidney and heart, and 35 kidney and pancreas, over a time period of up to 18 months. The JCV seroprevalence was shown as 76% at baseline, higher than reported previously (reviewed in [43]), and it increased continuously over time reaching 80.4%. 

A previous study [38] on 263 heart, kidney, liver, and pancreas transplant patients reported JCV DNA in the blood for 2.7% of kidney, 0.04% of kidney pancreas, 1.1% of heart, and 0.8% of liver grafts, which was transient in 69% of the episodes. The majority of JCV DNAemic episodes were subclinical (61.5%). Only 5 patients (38.5%) had clinical symptoms at the time of JCV DNAemia, one out of which had biopsy-proven acute heart rejection, whereas the other four patients had fatigue, lethargy, dyspnea, or tremors. No patients developed manifestations of PML. Since the overall JCV DNAemia rate was 5%, the authors remarked that JCV infection is a rare complication after SOT. JCV DNAemia was more common in kidney and/or pancreas transplant patients, possibly reflecting the reactivation of donor-derived JCV, which also persists in the kidneys [38]. These data were confirmed by Kamar and colleagues [44] that showed a higher JCV viremia in kidney (5.5%) than in liver and heart rituximab-treated transplant patients.

Regarding JCV infections in lung transplant recipients, Thomas and colleagues [45] tested urine samples for the presence of JCV with conventional PCR, demonstrating a positivity rate of 24% in at least one urine specimens. Mean viral load analyzed with q-PCR was 5 × 10^5^ copies/mL. However, there was no significant association between the immunosuppressive regiment and PyV infection. In fact, the prevalence of chronic graft dysfunction was 42% and 53% in patients who shed JCV or not, respectively. In addition, there was no significant correlation between urinary viral load and the patient's age and sex, the patient's immunosuppressive regimen, and the number of month after transplantation.

Although the different experimental approaches and the various results reported by the analyzed studies, they all agree that a strict attention should be paid to monitoring JCV infection, especially during the first 24 months following transplantation. In fact, even if JCV replication was mostly silent, it was not ruled out the hypothesis that it could be associated with certain clinical syndromes, as reported in Section 4.

## 4. JCV and Kidney Transplantation: The Virus-Associated Diseases

Infection by JCV has been observed in renal allograft recipients as both nephropathy and/or PML. PML occurs rarely in renal transplant patients and it is typically caused by JCV with high levels of viral genome found in the CSF (reviewed in [46]). However, recent reports suggest that another PyV, BKV, can also cause a PML-like disease [47, 48]. 

Renal transplant recipients have the highest risk of developing polyomavirus associated nephropathy (PyVAN) in comparison to other organ recipients because of the presence of ongoing graft injury due to drug toxicity, rejection episodes, cold ischemia, and donor/recipients HLA mismatch [49–52]. PyVAN with graft dysfunction and premature graft loss has been markedly increased since the 1990s [53, 54]; therefore, a pathogenic potential of JCV should be taken into account. In contrast to the closely related BKV, to date, only few cases of nephropathy have been attributed to JCV [55–59]. Low level of JCV replication and shedding are common in immunocompetent individuals [60, 61] but surprisingly the incidence of asymptomatic viruria is not increased in renal allograft recipients [62, 63]. This suggests that immunosuppressive state is not as strictly related to the development of PyVAN as it is for BKV [64, 65]. In addition, the immunosuppressive regimen does not play any important role and, once JCV PyVAN has been established, the reduction of immunosuppression has a controversial impact on the clinical course [66]. However, a profound immunosuppressive state is required for a pathological and potentially threatening JCV replication. In fact, patients with PML have significant JCV viruria and PML and JCV PyVAN have been reported to occur concurrently [58, 67–70]. This raises the question whether anti-CD20 biological therapy with rituximab in kidney transplant recipients is potentially cumbersome, because of a rapid depletion of pre-B and mature B cells that lasts for at least six months upon its administration. Our recent report in a small cohort of pediatric kidney transplant recipients showed that rituximab treatment had no effect on susceptibility to JCV replication [36]. These findings confirm some reports on adult population treated with either rituximab [71] or different immunomodulator drugs such as natalizumab [72, 73], where the risk of JCV new infection or reactivation was found inconsistent.

In a recent paper by Drachenberg and colleagues based on urine cytology and prospective protocol kidney biopsy in a cohort of hundred kidney transplant recipients, the incidence of JCV PyVAN was reported as low as 0.9% despite the fact that a significant proportion of the patients displayed JCV viruria or decoy cell shedding [22]. Interestingly, the majority of JCV PyVAN was diagnosed in patients with a normal renal function suggesting an apparently less aggressive or more protracted clinical course when compared with BKV PyVAN. This was recently confirmed by Cheng et al. in a larger cohort of kidney transplant recipients where the clinical outcome of JCV viruric patients was reported to be favorable up to five years following transplant [74]. Compared to non-JCV viruric patients, rejection rate, graft survival, and death-censored graft survival were lower and the patient survival was similar. Based on their results, they also suggested that JCV reactivation occurs in the native kidney on immunosuppression rather than in the donor-derived graft in contrast to BKV [52]. Another important difference between BKV PyVAN and JCV PyVAN is the strong association with viremia and the severity of histological pattern in the former [75]. On the contrary, low level of JCV viremia has been reported either in patients shedding large amounts of JCV in urine or in patients with parenchymal involvement and this may be related to fundamental differences between BKV and JCV biology, which remain presently unexplained [26, 38].

## 5. Conclusion

In conclusion, very few studies have been published regarding the replication of JCV in transplant patients. However, it is clear that JCV PyVAN is a unique clinical entity that needs to be differentiated from BKV PyVAN. This requires viral typing methods that are not widely available and this should account for an underestimation of its incidence in kidney transplant recipients. However, the protracted and nonaggressive clinical course of the disease and the favorable outcome should be considered once this form of PyVAN is diagnosed. Thus, monitoring of JCV infection, especially during the first 24 months after transplantation, is recommended.

## Figures and Tables

**Table 1:** Studies on JCV and kidney transplantation.

Subjects (*n*)	Sample	JCV (+)/subjects (%)	Pathological correlation	Reference
KTx: 103	Urine Blood	23/103 (22.3%) 0/103 (0%)	None	[21]
Controls: 23	Urine Blood	8/23 (34.7%) 0/23 (0%)		

KTx: 103	Urine Blood	28/103 (27.2%) 15/103 (14.2%)	None	[22]
Controls: 0	/	/		

KTx: 76	Urine Blood	12/76 (15.8%) 0/76 (0%)	None	[23]
Controls: 0	/	/		

KTx: 68	Urine	14/68 (21%)	None	[24]
Controls: 0	/	/		

KTx: 60	Urine	24/60 (40%)	None	[25]
Controls: 60	Urine	11/60 (18.3%)		

KTx: 186	Urine	33/186 (17.8%)	None	[26]
Controls: 0	/	/		

KTx: 25	Urine Serum	6/25 (24%) 6/25 (24%)	None	[21]
Controls: 20	Urine	3/20 (15%)		

KTx: 52	Urine	8/52 (15.4%)	None	[27]
Controls: 30	Urine	0/30 (0%)		

KTx: 59	Urine	2/59 (3.4%)		
CRD: 102	Urine	4/102 (3.9%)	None	[28]
Controls: 134	Urine	27/134 (20.1%)		

KTx: 103	Blood	7/103 (6.8%)	None	[29]
Controls: 0	/	/		

KTx: 30	Urine	7/30 (23.3%)	None	[30]
Controls: 30	Urine	7/30 (23.3%)		

KTx: 30	Urine	5/30 (16.7%)	None	[31]
Controls: 0	/	/		

KTx: kidney transplant recipients; CRD: chronic renal diseases.
